# Appropriate use of indwelling urethra catheters in hospitalized patients: results of a multicentre prevalence study

**DOI:** 10.1186/1471-2490-12-25

**Published:** 2012-09-06

**Authors:** Irálice AV Jansen, Titia EM Hopmans, Jan C Wille, Peterhans J van den Broek, Tjallie II van der Kooi, Birgit HB van Benthem

**Affiliations:** 1 Centre for Infectious Disease Control, National Institute for Public Health and the Environment (RIVM), PO box 1 (postbak 75), 3720 BA, Bilthoven, The Netherlands; 2 Department of Infectious Diseases, Leiden University Medical Centre, Leiden, The Netherlands

**Keywords:** Catheterization, Hospitalized patients, Inappropriate use, Prevalence study, Urethra catheters.

## Abstract

**Background:**

Although indwelling urethra catheterization is a medical intervention with well-defined risks, studies show that approximately 14–38% of the indwelling urethra catheters (IUCs) are placed without a specific medical indication. In this paper we describe the prevalence of IUCs, including their inappropriate use in the Netherlands. We also determine factors associated with inappropriate use of IUCs in hospitalized patients.

**Methods:**

In 28 Dutch hospitals, prevalence surveys were performed biannually in 2009 and 2010 within the PREZIES-network. All patients admitted to a participating hospital and who had an IUC in place at the day of the survey were included. Pre-determined criteria were used to categorize the indication for catheterization as appropriate or inappropriate.

**Results:**

A total of 14,252 patients was included and 3020 (21.2%) of them had an IUC (range hospitals 13.4-27.3). Initial catheter placement was inappropriate in 5.2% of patients and 7.5% patients had an inappropriate indication at the day of the survey. In multivariate analyses inappropriate catheter use at the time of placement was associated with female sex, older age, admission on a non-intensive care ward, and not having had surgery. Inappropriate catheter use at the time of survey showed comparable associated factors.

**Conclusions:**

Although lower than in many other countries, inappropriate use of IUC is present in Dutch hospitals. To reduce the inappropriate use of IUCs, recommended components of care (bundle for UTI), including daily revision and registration of the indication for catheterization, should be introduced for all patients with an IUC. Additionally, an education and awareness campaign about appropriate indications for IUC should be available.

## Background

Although indwelling urethra catheterization is a medical intervention with well-defined risks, such as catheter-associated urinary tract infection (CA-UTI), studies show that in hospitalized patients approximately 14–38% of the indwelling urethra catheters (IUCs) are placed without a specific medical indication [1-4]. Placement of urethra catheters is often uncomfortable [5]. Previous studies showed that up to 80% of all nosocomial UTI are related to the use of urethra catheters [6-9]. Moreover, their use has been associated with antibiotic use, morbidity, additional hospital costs, and mortality [1,7,10,11]. Despite these known disadvantages, IUCs are frequently used without an appropriate indication such as acute urinary retention. In the last years, much attention has been paid to the appropriate and inappropriate indications for IUCs [1,2,4]. Guidelines for catheterization and recommended components of care (e.g. bundle for UTI) were introduced aimed at reducing catheter-related complications [12-14]. In order to reduce the placement of IUCs without a specific medical indication, it is important to determine which factors are associated with the use of IUCs based on an inappropriate indication.

In the Netherlands, the voluntary surveillance system “Prevention of Nosocomial Infections through Surveillance” (PREZIES) was initiated in 1996 to monitor several nosocomial infections. In 2009, it was extended with a prevalence survey to study the use of IUCs. PREZIES is a collaboration between participating hospitals and the National Institute for Public Health and the Environment (RIVM). We use PREZIES data to describe the prevalence of IUCs, including its inappropriate use, and to determine which characteristics are associated with the inappropriate use of IUCs in hospitalized patients.

## Methods

### Study population and study procedure

Since 2007, national prevalence surveys of nosocomial infections are performed biannually in March and October within the PREZIES-network ( http://www.prezies.nl). From 2009 onwards, an optional theme “surveillance of IUCs” was integrated into the prevalence survey. In total, 4 prevalence surveys including the theme IUCs were performed in 2009 and 2010. All patients admitted to a participating hospital and who had an IUC in place at the day of the survey were included. Patients with a catheter in place at admission were not excluded. In 2009, only patients aged 1 year or older were included. Patients in day-care, psychiatric, and haemodialysis wards were excluded. Trained infection control professionals (ICP) or nurses at the hospital wards that are part of the PREZIES-network collected data. At the day of the survey, general data, catheter-related data, data on antibiotic use, and UTI data were collected for each patient using a standardized case record form. General variables included age, gender, medical speciality, medical ward (intensive care unit (ICU) vs. non-ICU), and previous surgery during current admission (yes vs. no). Catheter-related variables included the use of IUC and the indication for catheterization, both at the time of initial placement and at the time of the prevalence survey. In addition, information on the use of antibiotics and on symptomatic nosocomial UTI (and whether the latter was catheter-related) were recorded. Symptomatic UTI were measured and were classified as catheter-related if an IUC was in situ during (part of) the last 7 days before diagnosing a symptomatic UTI. Asymptomatic UTI were not measured in this study as they greatly depend on the local culture policy. Privacy of patients was provided by decoding all data according to the requirements of the existing privacy regulations in the Netherlands.

Pre-determined criteria, based on guidelines of the Dutch Working Group on Infection Prevention and revised by a multidisciplinary team, were used to categorize the indication for catheterization as appropriate or inappropriate (Table 1) [12]. The multidisciplinary team consisted of an urologist, an infectiologist, a ICP, and a member of the Dutch Working Group on Infection Prevention. Based on the recommendations given by this team, we added the categories other proper indication and other improper indication for IUCs to the list of indications for catheterization. The ICP or nurses categorized the indications for catheterization, at both the time of initial placement and at the time of the prevalence survey, according to Table 1. Patient records, information from ward staff and the local guidelines for the use of catheters were used to decide whether the indication was appropriate. Patients with an unavailable indication for catheterization in the medical record were discussed with the treating physician, specialist, or nurse. If there was insufficient information on the indication for catheterization, judgment of the appropriateness of the catheterization was not possible and the indication was coded as unknown. We deemed (in)appropriate indication for catheterization as (in)appropriate catheter use.

**Table 1:** **Indications for use of an indwelling urethra catheter**^**a**^

***Appropriate indications***
Urinary retention or bladder outlet obstruction
Close monitoring of urine output under non-operative conditions (e.g. incapable patient)
Pre- or post-operative use with a duration conform protocols
Neurogenic (overflow) bladder
Urinary incontinence in the presence of open sacral or perineal wounds
Administration of medication into the bladder / bladder flush during bleeding
Palliative care for terminal ill patients
Other proper indication, based on local hospital guidelines
***Inappropriate indications***
Urinary incontinence without open sacral or perineal wounds
Ulcer prevention
No real need for monitoring of urine output
Pre- or post-operative use with a duration not conform protocols
Other improper indication, based on local hospital guidelines

^a^ Based on guidelines of the Dutch Working Group Infection Prevention.

### Statistical analyses

Differences between the patients with an IUC and the patients without an IUC were tested using the Mann–Whitney-U test and the Chi-square test. The prevalence of IUCs was determined and the percentages of inappropriate catheter use at the time of initial placement and at the time of the prevalence survey were calculated. The (in)appropriateness of urethra catheters that were not judged because of insufficient information were identified as missing values. In sensitivity analysis, we categorized these missing values as appropriate and subsequently inappropriate to obtain a range for the occurrence of inappropriate IUC use. Furthermore, we determined the percentage of patients who had an inappropriate indication for catheterization at initial placement as well as an inappropriate indication for catheterization at the day of the prevalence survey.

We identified risk factors for inappropriate indwelling urethra catheterization at the time of initial placement and at the time of the prevalence survey using multilevel logistic regression models to account for intra-hospital correlations. All variables with a *p*-value ≤0.10 in univariate analysis were entered into the multivariate analyses. We built multivariate models for both time points using manual backward-stepwise procedure. A *p*-value ≤0.05 was considered statistically significant. As the missing values concerning the (in)appropriateness of catheter use may affect the results, we conducted a sensitivity analysis in which we repeated the univariate and multivariate analyses including these missing values categorized as appropriate and subsequently inappropriate. Statistical analyses were performed using the statistical software packages SAS version 9.2 and SPSS version 18.0.

## Results and discussion

In total 14,252 patients were included from 28 hospitals. One of the 28 participating hospitals was a university medical centre and the others were general acute care hospitals. The median age of the patients was 67.3 years (inter-quartile range (IQR) 51.4–78.1), 52.4% were female, 4.9% were admitted to an ICU ward, 32.3% had had surgery and 29.1% received one or more antibiotics on the day of the prevalence survey.

### Use of indwelling urethra catheters

Of those 14,252 included patients, 3020 (21.2%) had an IUC in place on the day of the survey (range hospitals: 13.4–27.3%) (Table 2). Patients with an IUC were older than patients without an IUC (73.1 years vs. 65.4 years; *P* < 0.001), underwent surgery more often (52.2% vs. 27.0%; *P* < 0.001), were admitted to an ICU ward more often (17.1% vs. 1.6%; *P* < 0.001), and received antibiotics more often (42.7% vs. 25.5%; *P* < 0.001). The gender distribution was equal between the two groups (female, 51.4% vs. 52.7%, *P* = 0.19).

**Table 2:** Characteristics of hospitalized patients with an indwelling urethra catheter


Number of patients	3 020
Median age (IQR)	73.1 (62.2– 81.1)
Female, %	51.4
Medical specialty, %	
Cardiology	8.9
Cardiothoracic surgery	4.0
Gastrointestinal and liver diseases	1.6
Geriatrics	1.9
Internal medicine	13.3
Neurology	6.6
Neurosurgery	2.1
Obstetrics	6.2
and gynaecology	
Oncology	1.9
Orthopaedics	10.9
Respiratory	7.1
Surgery	22.3
Urology	8.5
Unknown or different^a^	4.7
Medical ward, %	
ICU	17.1
Non-ICU	82.9
Surgery^b^	
Yes	52.2
No	47.8
Antibiotic use	
Yes	42.7
No	57.3

**Figure 1:**
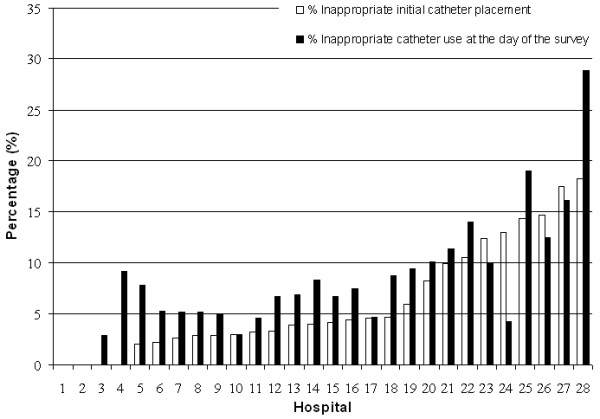
Percentages of inappropriate use of indwelling urethra catheters, at initial placement and at the day of the prevalence survey, per hospital in PREZIES.

Of the 3020 patients with an IUC, 95 (3.1%) patients had a symptomatic nosocomial UTI, of which 89 (93.7%) were catheter-related. Indication at the time of placement of an IUC and at the time of the prevalence survey could not be defined in 152 (5.0%) and 178 patients (5.9%), respectively. Initial catheter placement was judged inappropriate in 148 of 2868 patients (5.2%), and 214 of 2842 patients (7.5%) had an inappropriate indication at the day of the survey. Figure 1 shows the variation in the percentages of inappropriate catheter use in the different hospitals. In the majority of the hospitals (n = 21), the percentage of inappropriate catheter use at initial placement was lower than the percentage of inappropriate catheter use at the day of the prevalence survey. Four hospitals showed the opposite and in two hospitals there was no inappropriate catheter use. In sensitivity analysis inappropriate use at initial placement varied from 4.9% when we categorized the missing values of indication for catheterization as appropriate and to 9.9% when we categorized the missing values as inappropriate. The range for inappropriate catheter use at the time of the survey varied between 7.1 and 13.0% using the same methodology.

When looking at catheterization within patients, 122 of 2811 patients (4.3%) had an inappropriate indication for catheterization at initial placement and an inappropriate catheterization at the time of the survey. Eighty-four of 2811 patients (3.0%) had an appropriate indication for catheterization at initial placement, but subsequent an inappropriate indication for catheterization at the time of the prevalence survey. Urinary incontinence without open sacral or perineal wounds was one of the most common inappropriate indications for both placement (35.8%) and catheterization at the time of the survey (28.0%) (Table 3). 42.6% of the improper indications at initial placement and the 48.1% of the improper indication on the survey day were based on other unspecified improper indications mentioned in the local guideline.

**Table 3:** Indications for inappropriate use of indwelling urethra catheters at initial placement and the survey day

	**Initial placement (%)**	**Prevalence survey (%)**
**Inappropriate indication (n)**	148	214
Urinary incontinence without open sacral or perineal wounds (n)	35.8 (53)	28.0 (60)
Ulcer prevention (n)	10.8 (16)	5.1 (11)
No real need for monitoring of urine output (n)	3.4 (5)	2.3 (5)
Pre- or post-operative use with a duration not conform protocols (n)	7.4 (11)	16.4 (35)
Other improper indication, based on local hospital guidelines (n)	42.6 (63)	48.1 (103)
n, number.		

### Factors associated with inappropriate use of indwelling urethra catheter

The factors associated with inappropriate use of IUC at initial placement in univariate analyses were: age, gender, medical ward, and surgery. In multivariate analyses, inappropriate catheter use at the time of placement was independently associated with female sex (odds ratio (OR) 1.73, 95% confidence interval (CI) 1.17–2.56), older age (OR 1.30, 95% CI 1.12–1.52), admission on a non-ICU ward (OR 4.80, 95% CI 1.84–12.52) and not having had surgery (OR 2.40, 95% CI 1.59–3.62) (Table 4). Inappropriate catheter use at the time of the survey showed comparable associated factors in both the univariate and multivariate models (Table 4). Furthermore, sensitivity analyses that included the missing values showed the same direction effects of the associated factors at both time points.

**Table 4:** Multilevel logistic regression analyses for inappropriate use of indwelling urethra catheters in hospitalized patients

	**Initial placement**		**Prevalence survey**	
	**Univariate**	**Multivariate**	**Univariate**	**Multivariate**
**Risk factor**	OR (95% CI)	OR (95% CI)	OR (95% CI)	OR (95% CI)
Age^a^	1.46 (1.25-1.72)**	1.30 (1.12-1.52)*	1.38 (1.21-1.56)**	1.27 (1.12-1.43)**
Gender				
Male	1.00*	1.00*	1.00*	1.00*
Female	1.86 (1.26-2.73)	1.73 (1.17-2.56)	1.58 (1.15-2.17)	1.45 (1.05-2.00)
Medical ward				
ICU	1.00**	1.00*	1.00**	1.00**
non-ICU	6.16 (2.5-16.67)	4.80 (1.84-12.52)	10.0 (3.70-25.00)	7.85 (3.04-20.25)
Surgery				
Yes	1.00**	1.00**	1.00**	1.00*
No	2.78 (1.89-4.17)	2.40 (1.59-3.62)	1.82 (1.33-2.50)	1.56 (1.12-2.17)

CI, confidence interval; ICU, intensive care unit; OR, odds ratio.

^a^ incremental increase by 10years.

*P value, <0.05; **P value <0.001.

In this study, the overall mean prevalence of IUCs in hospitalized patients was 21.2%. This finding is in line with other papers reporting prevalence of urethra catheters between 15 and 25% for patients in general hospitals [13,15]. In contrast with our prevalence, a study of Apisarnthanarak et al. reported a lower prevalence of IUCs [2]. The difference between this study and our might be explained by differences in patient populations, possibly due to different inclusion criteria, and differences in hospitals characteristics and guidelines.

Compared to other studies, the patients who had an IUC in our study had a low percentage of nosocomial UTI. It is known that the majority of the CA-UTIs are asymptomatic [16,17]. Our low percentage of UTI might be explained by the exclusion of the asymptomatic UTIs. Nevertheless, the majority of the symptomatic UTIs we found (93.7%) were catheter-related, confirming that the use of IUC is associated with UTI.

At initial placement 5.2% of the IUC were considered inappropriate, whereas on the day of the prevalence survey 7.5% of IUC were inappropriate. Both mean percentages of incorrect catheter use are lower than in many other studies investigating the (in)appropriateness of IUCs [3,4,8,18,19]. It could be that the missing values of indication for catheterization represented a specific group in which all indications were inappropriate. In that case the percentage of inappropriate catheter use would be underestimated in our study. However, there were no differences in the patient-related and hospital-related variables between the patients without a judgment of the urethra catheter and the others in whom the indication for catheterization was available (data not shown). Furthermore, when we categorized the missing values of indication for catheterization as inappropriate, the maximum inappropriate catheter use at initial placement and on the day of the prevalence survey turned out to be 9.9% and 13.0%, respectively. Both percentages were still low, but in line with another study who found a similar low percentage of inappropriate catheter use.^1^

We demonstrated that the percentage of inappropriate initial placement of a urethra catheter was lower than the percentage of inappropriate catheterization at the day of survey. Previous studies also found that initial indication for the placement of an IUC was justified in a significantly greater proportion of observations compared to the indication for continued catheterization [3,19]. Indication for insertion of a urethra catheter is often considered. However once inserted, catheters tend to remain in place after the appropriate indication for their usage has ended, possibly resulting in catheter-related complications. Approximately 26% of patients who have an IUC in place for 2–10 days will develop bacteriuria, and 25% of those patients will develop a CA-UTI. [13] In addition, antimicrobial resistance among urinary pathogens is an increasing problem [13]. Therefore, much attention has to be paid to the appropriate indications for catheterization and specifically on the daily indication for prolonged catheterization.

The use of IUCs based on inappropriate indication was associated with patient characteristics as well as general factors. Women and older patients are at increased risk for inappropriate catheterization. These associated factors were comparable with those found by one of the few studies on risk factors for inappropriate catheterization [20]. In addition, we found that non-surgical patients and non-ICU patients were also at risk for inappropriate use. Apparently, the evaluation of the indication for catheterization for patients undergoing surgery or for patients admitted to an ICU is better. In univariate analysis medical specialty as independent factor, patients admitted to a urology ward had more often an appropriate indication for catheterization than patients admitted to other wards (data not shown). Risk factors for inappropriate catheter utilization allow hospitals to target quality improvement projects, for example training sessions for non-surgical wards staff.

It is possible that there were differences between hospitals in the implementation of the surveillance-protocol. In order to control for this possible inter-hospital variation, workshops explaining the protocol, including the study procedure, were organized for all the ICP involved in this survey.

## Conclusions

Inappropriate use of IUC is present in Dutch hospitals, although the prevalence is lower than in many other countries. Women, older patients and non-surgical patients are at higher risk for catheterization without a proper medical indication. To reduce the inappropriate use of IUCs, recommended components of care (bundle for UTI) should be introduced for all patients with a urethra catheter. This UTI bundle, aimed at reducing catheter-related complications, should include daily observation and registration of the indication for catheterization. Additionally, an education and awareness campaign about appropriate indications for IUCs should be available, especially for the medical staff on the non intensive care wards.

## Abbreviations

IUCs: Indwelling Urethra Catheters; CA-UTI: Catheter-associated Urinary Tract Infection; UTI: Urinary Tract Infection; PREZIES: Prevention of Nosocomial Infections through Surveillance; IQR: Inter-Quartile Range; ICP: Infection Control Professional; ICU: Intensive Care Unit; OR: Odds Ratio; CI: Confidence Interval.

## Competing interests

None declared.

## Authors' contributions

IJ performed the statistical analysis and drafted the manuscript. TH participated in the design and coordination of the study and helped to draft the manuscript. JW participated in the design and coordination of the study and helped to draft the manuscript. PvdB critically reviewed the draft manuscript and participated in the design of the study. TvdK participated in the statistical analysis and helped to draft the manuscript. BvB participated in the design of the study, supervised the statistical analysis and critically reviewed the draft manuscript. All authors read and approved the final manuscript.

## Pre-publication history

The pre-publication history for this paper can be accessed here:

http://www.biomedcentral.com/1471-2490/12/25/prepub
